# A Validation of an Intelligent Decision-Making Support System for the Nutrition Diagnosis of Bariatric Surgery Patients

**DOI:** 10.2196/medinform.2984

**Published:** 2014-07-08

**Authors:** Magda RR Cruz, Cristina Martins, João Dias, José S Pinto

**Affiliations:** ^1^Pontifical Catholic University of Paraná (PUCPR)CuritibaBrazil; ^2^Cristina Martins Institute and Kidney FoundationCuritibaBrazil; ^3^Department of Electrical EngineeringFederal University of ParanaCuritibaBrazil; ^4^Science, Management, and Information TechnologyFederal University of ParanáCuritibaBrazil

**Keywords:** bariatric surgery, nutrition diagnosis, artificial intelligence, Bayesian networks, decision-making, support system

## Abstract

**Background:**

Bariatric surgery is an important method for treatment of morbid obesity. It is known that significant nutritional deficiencies might occur after surgery, such as, calorie-protein malnutrition, iron deficiency anemia, and lack of vitamin B12, thiamine, and folic acid.

**Objective:**

The objective of our study was to validate a computerized intelligent decision support system that suggests nutritional diagnoses of patients submitted to bariatric surgery.

**Methods:**

There were fifteen clinical cases that were developed and sent to three dietitians in order to evaluate and define a nutritional diagnosis. After this step, the cases were sent to four bariatric surgery expert dietitians who were aiming to collaborate on a gold standard. The nutritional diagnosis was to be defined individually, and any disagreements were solved through a consensus. The final result was used as the gold standard. Bayesian networks were used to implement the system, and database training was done with Shell Netica. For the system validation, a similar answer rate was calculated, as well as the specificity and sensibility. Receiver operating characteristic (ROC) curves were projected to each nutritional diagnosis.

**Results:**

Among the four experts, the rate of similar answers found was 80% (48/60) to 93% (56/60), depending on the nutritional diagnosis. The rate of similar answers of the system, compared to the gold standard, was 100% (60/60). The system sensibility and specificity were 95.0%. The ROC curves projection showed that the system was able to represent the expert knowledge (gold standard), and to help them in their daily tasks.

**Conclusions:**

The system that was developed was validated to be used by health care professionals for decision-making support in their nutritional diagnosis of patients submitted to bariatric surgery.

## Introduction

### Nutrition and Bariatric Surgery

Morbid obesity causes a number of health issues, explaining why, in certain situations, some aggressive treatments may be used, for instance, bariatric surgery. The surgical procedure is indicated when the patient presents a body mass index over 40 kg/m^2^, or when it is situated between 35 and 40 kg/m^2^ and also presents some associated disease, such as, diabetes, dyslipidemias, cardiovascular and cerebrovascular diseases, sleep apnea, joint disease, and orthopedic disease, among others [1]. It is estimated that one million bariatric surgeries will be performed in the next few years in the United States alone [2]. Therefore, the concern related to the nutritional changes in the long term in these patients is highly important [3-7]. Furthermore, the need for individualized management of patients with obesity is evident [3-7]. Thus, the health professional concern related to some special nutritional care is comprehensible, particularly in relation to eating in the pre and post operatory in bariatric surgeries.

Some of the most common nutritional deficiencies include iron, vitamin B12, folate, thiamine, and protein after bariatric surgery [2,5,8-11]. Severe consequences can be expected when they are not prevented or treated early.

### The Nutrition Care Process

The Nutrition Care Process consists of four steps: (1) nutrition assessment, (2) nutrition diagnosis, (3) nutrition intervention, and (4) nutrition monitoring and evaluation. The nutrition diagnosis, the second step of the Nutrition Care Process, is the identification and record that describes an actual occurrence, risk of, or potential for developing a nutritional problem [12].

The results from the use of this technology, which are achieved by computing beyond the nutritional science knowledge, are important in order to help in detecting nutritional deficiencies. The information technology in the field of health has tools and instruments that may support the administrative organization in patient service. These tools and instruments are able to capture, store, and process information, and may offer some diagnosis suggestions, therapeutic orientation, and access to information [13]. The specialized systems are very helpful for the health professionals. In particular, there is the so-called Decision Support System (DSS).

These programs are used to help the professionals to define the diagnosis through artificial intelligence. A Bayesian network (BN) is the technique used in the formulation of DSS. It is able to represent the uncertainty in knowledge through the Bayes’ Theorem. In this case, the necessary data for the model is collected through published statistical studies and/or through specialist consultation [14]. The Bayes’ Theorem calculates the probabilities in each diagnosis, given a set of pre existing information [14]. The fact that it can work with uncertainty through probability makes it the most significant technique to be used in the health field.

### Aim of the Study

The aim in this study is to validate a DSS that will help the nutritional diagnosis for bariatric surgery patients through the development of a protocol created by experts in the field, given the large number of surgeries, the long term nutritional risks, the small amount of specialists in the field, and the absence of a specific computer system.

## Methods

### The Selection Process

The prevalence of each nutritional diagnosis has different probabilities, depending on the bariatric surgery technique used. Therefore, only patients submitted to the surgical technique Roux-en-Y gastric bypass were selected for this study. These diagnoses are currently considered the gold standards [15].

### The First Stage

The first stage of the study comprised the knowledge base building. There were two resources that were used in order to do so: (1) scientific studies published in internationally recognized journals, in addition to important studies in the fields of nutrition and medicine; and (2) consultations with nutrition specialists. From these sources, the major nutritional deficiencies presented in the post operatory were verified [1,2,9-11], the average weight loss found in patients was noted [16], the main signs and symptoms in patients were described [8,11,17], and the definitions of the techniques used in the nutritional assessment were identified [18,19].

The results from this stage indicated that a specialized module of nutritional diagnosis should consider gender, age, surgery time, biochemical markers (hemoglobin, hematocrit, mean corpuscular volume, serum albumin, ferritin, vitamin B12 and folic acid), food intake, and physical signs and symptoms of nutrient deficiency. This study opted for classifying them as high, low, or normal, according to the usual standard references, due to a wide range of techniques to measure the selected biochemical markers. The analysis of the number of food portions consumed for the food intake evaluation was based on the Food Guide Pyramid [19]. The reference was 1600 kcal, which is the minimum amount recommended for a suitable macro and micronutrients intake. The physical signs (hair loss, changes in nails and skin, paleness) and symptoms (weakness, paresthesia, vomiting, diarrhea, blood loss) are derived from the subjectivity of professionals who qualify the information before it is used by the system. Because the data on dietary intake and the signs and symptoms are subjective, BNs have been selected for the representation of knowledge. The technique considers the evidence presented for the calculation of the disease probability in case it happens, and allows that the subjectivity or the uncertainty element of information be considered. Last, the standard nutritional diagnoses were protein-energy malnutrition, iron deficiency anemia, vitamin B12 deficiency, folic acid deficiency, and thiamine deficiency. Additionally, tools to identify risks to develop all these deficiencies were created.

From the tools mentioned above, a study of the variables was carried on, considering each nutritional diagnosis for each patient. For instance, for a patient with iron deficiency, all the signs, symptoms, dietary intake, and biochemical markers indicated from the literature were analyzed. All of the information that either caused doubts or did not help in the diagnosis conclusion were excluded for not being considered decisive in the decision support. In other words, only the variables that influenced in the diagnosis decision were kept in the study.

After assembling the qualitative part of the network (inclusive and exclusive definition of variables), probabilistic values ​​were assigned for each of them, as described in the literature. Thus, the quantitative part of the network was originated. As there was no availability of a database containing all the variables and attributes required to work, the use of literature and discussion with experts were chosen. For each nutritional diagnosis, the probability of the event in the presence or absence of the disease, or the risk of the development of each one of the variables, was considered.

### The Technology Used

The technique implementation of the BN was performed with the aid of Shell Netica. It has the infrastructure to develop expert systems within a pre built interface. The program Netica is composed by Netica Application and the Netica Application program interface (API). The Netica Application is a graphical interface that permits you to view the knowledge base in a network. The Netica API is the library of the program, written in C language, which is available on a website [20] on the Internet.

### Preliminary System Evaluation

In the first step of the nutritional diagnosis support system validation, fifteen case studies were developed and elaborated on by two nutritionists; one was an expert in morbid obesity, and the other one was not. All the case studies were sent to four expert nutritionists in the field of nutrition in order to get evaluations and diagnosis reports from them. A standard diagnosis list was attached to the case studies. It was also requested that the evaluators suggest changes in the developed clinical cases and in the diagnostic proposal. The four experts' answers were compared to those given by the system, and the experts' answers were revised based on this evaluation. Thereby, a proposal for the nutritional diagnosis support system was presented called DSS Diagnosis Nutrition 1. This contained the case studies reviewed, according to the nutritionists’ opinion.

### Gold Standard Development

The experts were selected for the gold standard development based on: (1) nutrition studies background, (2) over two years as a member of the multidisciplinary team for the treatment of patients submitted to the obesity surgery, and (3) if they have followed more than 300 patients in the post operatory. There were four experts that were selected according to these criteria. They received the fifteen case studies revised, and had to send diagnosis reports for each of them. The experts’ reports were compared among themselves. The disagreements were solved through consensus among the experts, resulting in the gold standard. This standard aimed to evaluate the system performance.

### System Validation Technique

The following analyses were performed for the final system validation: (1) comparison between the four experts’ success rates, the gold standard success rate, and between the system and the gold standard; (2) calculation of sensibility and specificity for each nutritional diagnosis; and (3) the receiver operator characteristic (ROC) curve construction for each diagnosis.

All the ethical principles in the Helsinki Declaration (2000) [21] were respected during the development of this study. There was no direct participation of human beings.

## Results

### Success Rate Between the Experts and the Gold Standard

The qualitative part of the BN was done considering the interrelation among the nutritional diagnosis and the signs, symptoms, food intake, and biochemical markers. As a result, five subnets were obtained, each one of them featuring a nutritional diagnosis: (1) vitamin B12 deficiency, (2) thiamine deficiency, (3) folic acid deficiency, (4) iron deficiency, and (5) malnutrition. The health professional could classify the patients’ diagnosis as “present”, “absent”, or “in risk” of developing it. At the same time, the four experts selected to build the gold standard diagnosis were questioned after assessing the fifteen clinical cases sent to them. That originated 60 answers per nutritional diagnosis. The experts’ diagnoses were compared to the gold standard, creating the experts assertiviness rate related to the gold standard (Table 1).

**Table 1:** Success rate for nutritional diagnosis between the four experts and the gold standard/ BN algorithm.

Cases	Iron deficiency anemia	Folate deficiency	B12 deficiency	Thiamine deficiency	Malnutrition
Number of success/total	54/60	56/60	48/60	52/60	55/60
Assertiveness (%)	90	93	80	87	92
Standard deviation	23	11	25	23	15

### Expert Disagreements

Vitamin B12 and thiamine deficiencies were the diagnoses that most presented disagreements among experts, followed by iron deficiency anemia. The values in Table 1 were 48, 52, and 54 assertiveness respectively, in a sample of 60 cases. In other words, the result was higher than that found among the four experts, which presented a variation between 80% (48/60) and 93% (56/60), according to the diagnosis. That showed that even though there are criteria for each nutritional diagnosis, the individual interpretation could make the task difficult.

The answers reported by the four experts were analyzed individually, causing greater disagreement in the definition of the diagnosis of the problem or presence of risk, thus, reinforcing the usefulness of the system to aid the diagnosis, either confirming the professional hypothesis or warning them of the disease risk.

### System Assessment in Relation to the Gold Standard

#### Success Rate of the System

The diagnoses reports from the system were compared to the reports from the gold standard in order to assess the performance of the system. The success rate found was 100% (60/60) for the case reports diagnosed. Taking as an example the first clinical case presented to the experts, the following situation was observed, the gold standard detected risk to the development of iron deficiency anemia, folic acid deficiency, thiamine deficiency, and malnutrition. None of the diagnoses were confirmed, and the presence or the risk of development of vitamin B12 deficiency was rejected. When the same data was input to the system, this presented values higher than 70.0% (70/100) of risk of development of folic acid deficiency, of vitamin B1 deficiency, and of malnutrition. The values were 100.0% (100/100) for iron deficiency anemia. The vitamin B12 deficiency, which was a diagnosis rejected by the gold standard, presented 0.11% (.11/100) chance of confirmation and 33.5% (33.5/100) of risk of development. The same happened to the other cases, thus proving that there was agreement between the reports provided by the system and the gold standard.

The percentage for the diagnosis and for the risk of development was very close in some cases, when each case was analyzed individually. For instance, in case 3 the patient presented 42.3% (42.3/100) of probability of confirmation of the diagnosis for anemia, and 56.3% (56.3/100) of probability of risk of development of anemia. The gold standard classified the patient as in risk of anemia, agreeing with the system.

#### Sensibility and Specificity for Each Nutritional Diagnosis and the Construction of the Receiver Operator Characteristic Curve

The Medicalc was used for the analysis of sensibility and specificity, determining the ability to discriminate among diagnoses through the ROC curve. There was a comparison between the reports from the system and those from the gold standard. The results are in Table 2.

**Table 2:** BN diagnosis test results.

Test assessment	Presence of risk or diagnosis x absence				
	Iron	Folic acid	B12	Thiamine	Malnutrition
Sensibility %	95.0	95.0	95.0	95.0	95.0
Specificity %	95.0	95.0	95.0	95.0	95.0
Area below the ROC curve	0.893	1.0	1.0	0.982	1.0
Standard error	0.088	0.0	0.0	0.038	0.0
95% confidence interval	0.627 - 0.986	0.78 - 1.0	0.78 - 1.0	0.751 - 1.0	0.78 - 1.0

#### Results of the Comparison

It was observed that the specificity and the sensibility of the system presented high levels (95.0%) for all the diagnoses. The results reflect the validation of its use. In other words, the system is able to represent the gold standard. Besides that, the confidence interval was well established and the standard error was low (0.0-0.088). The results also confirmed the agreement between the gold standard and the system.

Regarding the ROC curve, it was observed that for the folic acid deficiency, vitamin B12, and malnutrition, the system presented maximum performance (1.0).

The results found for iron deficiency anemia and for thiamine deficiency also indicated a good performance of the system. However, there was a small deviation in its projection, which represents the possibility of disagreement between the system and the gold standard. In the end, the analysis of the data showed in the ROC curve concluded that the system presented a good performance in the definition of each diagnosis, thus being able to be used in the aid of health professionals.

## Discussion

### Expert Diagnoses

The success rate from the developed system was higher than that found among the four experts. This result reflects the difficulty in the diagnosis definition by the specialists. The fact is comprehensible since the definition of a diagnosis involves different information, previous experiences, and many times, the use of common sense and intuition. The mental mechanisms and the processes of thinking used by a specialist to arrive at a diagnosis are still poorly understood. Many times there is a lack of consensus among specialists, in some fields [22,23]. Furthermore, as the nutritional following-up of patients who were submitted to a bariatric surgery is still something recent, the disagreement among professionals may be common. In this study, the development of a gold standard by nutrition specialists was essential. Not only due to the need of a reference, but also for creating a discussion and reflection regarding the diagnosis that each specialist had previously established. This discussion confirmed the need of a system that makes the professional think about other possibilities, before the final nutritional diagnosis.

### Decision Support Systems

There are not any other intelligent DSSs that have been developed specifically for the nutritional monitoring of patients undergoing bariatric surgery known by this group. Because of that, there was no chance of our system being compared to any other similar systems. Quick Medical Reference System is a system that helps in the diagnosis of many fields in medicine. It presents a success rate of 85% [24]. Our system presents a success rate of 100% (60/60) for the case reports diagnosed. Therefore, its good performance is confirmed as well as its indication of use.

The developed system in this study presents the possibility of working with the probability of risk/disease, versus the absence of nutritional risk, and enables the health professional not only to detect diseases, but also to detect the risk of developing them. Thus, it increases the possibility of prevention, of early treatment, or even a specific follow-up, therefore preventing a disease from progressing to more serious stages. Thus, it is expected that the system assists in the patients’ follow-ups, not only suggesting the nutritional diagnosis, but also preventing the major deficiencies that can occur post operatively in bariatric surgery.

Another extremely important factor that should be considered is the possibility of changing or adding variables to the system in the future, as the developments of new studies and the experts’/users’ opinions occur. This aspect facilitates the maintenance and improvement of the system’s performance. The inclusion of data to assist in the diagnosis of other nutritional deficiencies, and that are currently being researched, may enrich the system in the future. This is the situation of osteoporosis, which may occur in the late post operative period, or even the zinc deficiency, which is often mentioned, but rarely diagnosed in clinical practice.

### Conclusions

This study enabled the validation of a DSS to assist the health professional in the nutritional monitoring of patients submitted to bariatric surgery.

The aim has been achieved since the system was able to duplicate the reports issued by the gold standard, both in the presence of disease and in the risk of developing it. The construction of an elaborate knowledge base proves to be essential in obtaining results. The result of showing the probabilities of the patient having the disease or the risk of developing it, rather than just issuing reports with categorical outcomes (“yes” or “no”), increases the freedom of the professional making the decision. In other words, it does not make the diagnosis authoritative, but suggestive.

It can be affirmed, through this study, that BN is an effective tool in the duplication of expert knowledge when there are several factors with different probabilities of occurrence involved in the definition of a diagnosis. Therefore, its use is indicated in health care.

In conclusion, the information gathered while developing DSSs for nutritional diagnosis can facilitate health professionals’ tasks. The goal is not to replace professional work, but to help decision making. The intention is not to solve clinical cases, but to instigate critical thinking before the diagnostic decision.

## Abbreviations

API: application program interface
BN: Bayesian networks
DSS: Decision Support System
PUCPR: Catholic University of Paraná
ROC: receiver operator characteristic
